# 135 Evaluation of Autologous Skin Cell Suspension on Re-epithelialization and Repigmentation in Pre-clinical Models

**DOI:** 10.1093/jbcr/irae036.134

**Published:** 2024-04-17

**Authors:** Katie A Bush, Ghaidaa Kashgari, Sohail Jahid, Heather M Powell, Steven A Kahn

**Affiliations:** AVITA Medical, Marblehead, Massachusetts; AVITA Medical, Irvine, California; AVITA Medical, Valencia, California; The Ohio State University, Columbus, Ohio; MUSC, Charleston, SC; AVITA Medical, Marblehead, Massachusetts; AVITA Medical, Irvine, California; AVITA Medical, Valencia, California; The Ohio State University, Columbus, Ohio; MUSC, Charleston, SC; AVITA Medical, Marblehead, Massachusetts; AVITA Medical, Irvine, California; AVITA Medical, Valencia, California; The Ohio State University, Columbus, Ohio; MUSC, Charleston, SC; AVITA Medical, Marblehead, Massachusetts; AVITA Medical, Irvine, California; AVITA Medical, Valencia, California; The Ohio State University, Columbus, Ohio; MUSC, Charleston, SC; AVITA Medical, Marblehead, Massachusetts; AVITA Medical, Irvine, California; AVITA Medical, Valencia, California; The Ohio State University, Columbus, Ohio; MUSC, Charleston, SC

## Abstract

**Introduction:**

The autologous cell harvesting device prepares an autologous skin cell suspension (ASCS) from a thin skin sample at the point of care. This study reports on advanced cellular characteristics of ASCS and provides pre-clinical evidence of functional regeneration of the epidermis of full-thickness injuries including re-epithelialization, repigmentation, basement membrane formation, and barrier function.

**Methods:**

Autologous skin cell suspension was prepared from excised discarded healthy donor tissue. Cellular density, viability, phenotypes, apoptotic activity, and number of aggregates were analyzed over 10 donors. To evaluate functionality, two pre-clinical model systems were used. The first system utilized an in vitro model composed of a 3D living dermal equivalent containing viable collagen and fibroblasts. ASCS was applied to the dermal equivalent and cultured over 21 days. The second model was an in vivo model, using full-thickness excisional wounds in Yorkshire pigs. ASCS was prepared and applied onto the wound bed in combination with 3:1 meshed autograft. Healing outcomes were recorded through visual observations and histology was assessed for epidermal morphology (cell localization, stratification), re-epithelialization, proliferation, and basement membrane formation.

**Results:**

ASCS prepared from 4 cm2 donor skin had a mean of 2.41E6±1.03E6 total cells/ml and from 24 cm2 donor skin had a mean of 2.76E6±6.80E5 total cells/ml. The majority of cells found in ASCS were non-apoptotic single cells. Advanced phenotyping data suggests presence of basal keratinocytes and activated keratinocytes, both known to play a role in wound healing and epidermal regeneration.

The in vitro model demonstrated reconstruction of an epidermal layer with basement membrane formation, and localization of melanocytes and proliferative cells at the dermal-epidermal junction. Additionally, this model system demonstrated the functionality of the melanocytes, as pigmentation was macroscopically observed at 21 days.

Full-thickness wounds treated with ASCS+mSTSG in the porcine model successfully re-epithelialized, with a thin layer of epidermal cells present in the interstices of the expanded 3:1 mSTSG seen as early as 3 days post-wounding. At 6 days, average percent re-epithelialization in the interstices was 91%. The regenerated epidermis contained highly

**Conclusions:**

These pre-clinical models demonstrate the functionality of ASCS and ability to regenerate a functional epidermis when the appropriate environment and signaling cues are present.

**Applicability of Research to Practice:**

This data provides scientific evidence supporting clinical results obtained when ASCS is used in conjunction with a mSTSG for the treatment of full-thickness wounds.

